# High Dietary Phosphate Exacerbates and Acts Independently of Low Autophagy Activity in Pathological Cardiac Remodeling and Dysfunction

**DOI:** 10.3390/cells10040777

**Published:** 2021-04-01

**Authors:** Mingjun Shi, Sierra Shepard, Zhiyong Zhou, Jenny Maique, Olivia Seli, Orson W. Moe, Ming Chang Hu

**Affiliations:** 1 Charles and Jane Pak Center for Mineral Metabolism and Clinical Research, University of Texas Southwestern Medical Center, Dallas, TX 75390, USA; Mingjun.shi@utsouthwestern.edu (M.S.); Sierra.shepard@utsouthwestern.edu (S.S.); In_bud@ctgu.edu.cn (Z.Z.); Jenny.maique@gmail.com (J.M.); Olivia.seli@utsouthwestern.edu (O.S.); 2 Department of Internal Medicine, University of Texas Southwestern Medical Center, Dallas, TX 75390, USA; 3 Department of Physiology, University of Texas Southwestern Medical Center, Dallas, TX 75390, USA

**Keywords:** apoptosis, atg5, autophagy, cardiomyocyte, cardiomyopathy, phosphotoxicity

## Abstract

High phosphate contributes to uremic cardiomyopathy. Abnormal autophagy is associated with the development and progression of heart disease. What is unknown is the effects of phosphate on autophagy and whether the ill effects of phosphate on cardiomyocytes are mediated by low autophagy. High (2.0% *w*/*w*)-phosphate diet reduced LC3 puncta in cardiomyocytes and ratio of LC3 II/I and increased p62 protein, indicating that autophagy activity was suppressed. Mice with cardiomyocyte-specific deletion of autophagy-related protein 5 (*H-atg5*^−/−^) had reduced autophagy only in the heart, developed cardiac dysfunction with hypertrophy and fibrosis, and had a short lifespan. When *H-atg5*^−/−^ mice were fed a high-phosphate diet, they developed more apoptosis in cardiomyocytes, more severe cardiac remodeling, and shorter lifespan than normal phosphate-fed *H-atg5*^−/−^ mice, indicating that cardiac phosphotoxicity is imparted independently of atg5. In conclusion, although high phosphate suppresses autophagy, high phosphate and low autophagy independently trigger and additionally amplify cardiac remodeling and dysfunction.

## 1. Introduction

Although phosphate is an essential component of cell structures, such as DNA phospholipids and phosphoglycans, and numerous cellular activities, including energy metabolism (e.g., ATP production) and kinase-mediated regulation (e.g., phosphoproteins) [1,2], phosphate excess is associated with various human diseases, such as bone, soft tissue, and cardiac complications in chronic kidney disease (CKD) [3,4,5,6,7,8,9]. The biologic ill effect of high phosphate, collectively termed phosphotoxicity, on cardiovascular diseases is attracting more attention [6,8,10]. Higher serum phosphate levels at baseline are associated with high all-cause mortality in patients after nearly 5-year post-myocardial infarction [11] and higher incidence of cardiovascular disease in healthy subjects [12,13]. Higher serum phosphate levels are also correlated with vascular and endothelial dysfunction [14], vascular and valvular calcification [15], and greater left ventricular mass [16]. Control of serum phosphate by phosphate binders and/or dietary phosphate restriction ameliorates vascular calcification and cardiac fibrosis [17,18] and reduces mortality in uremic rodents [17,19,20,21] and, to some extent, in patients with CKD [22,23,24,25]. Serum phosphate levels inversely correlate with average lifespan in different animal species, including humans [26,27]. These data are compatible with a pathogenic role of high phosphate in aging and cardiovascular diseases [18,26,27]. However, the mechanism by which high phosphate leads to cardiac remodeling has not been elucidated.

Autophagy is an evolutionarily conserved catabolic pathway to degrade, process, and recycle cellular components, thereby maintaining cellular homeostasis and balancing sources of energy in response to a variety of insults and stress [28,29,30]. Numerous studies confirmed that impaired autophagy is closely associated with human disease, including aging [29,31,32] and pathological cardiac remodeling [33,34,35]. Autophagy-related protein 5 (atg5) is one component in the autophagy machinery involved in the extension of the phagophoric membrane and early stages of autophagosome formation [36,37,38]. Cardiac-specific deficiency of atg5 led to cardiac hypertrophy, left ventricular dilatation, and contractile dysfunction in adult mice [39]. Our previous study demonstrated that constitutively high autophagy attenuated cardiac fibrosis and prolonged lifespan in old mice and mice with premature aging [32], implying that the maintenance of autophagy is a homeostatic mechanism for preserving normal cardiac structure and function and may be particularly important for retarding cardiac remodeling in the elderly.

An association between phosphate level and autophagy activity has been reported. Autophagy activation was shown to protect against phosphate-induced target tissue damage [40]. A higher serum phosphate concentration and less autophagy were found in skeletal muscle in old mice compared to young mice, and low autophagy in cultured myoblast cells was induced with high-phosphate media compared to normal phosphate [41]. Additionally, mice fed a high-phosphate diet had an accumulation of p62 [31,42], suggesting impaired autophagy activity. However, whether aberrant autophagy activity mediates phosphate-induced cardiac remodeling is not known.

To examine the molecular mechanism of how high phosphate affects cardiac dysfunction and pathologic remodeling, we first examined the effect of high dietary phosphate on the autophagy activity in the heart. Next, we explored the impact of the deletion of atg5 in cardiomyocytes on myocardial hypertrophy and cardiac fibrosis. Finally, to test the model of tandem events of phosphate loading–decreased autophagy–cardiac remodeling, we examined for additive effects of high dietary phosphate and myocardial atg5 deficiency on cardiomyopathy.

## 2. Materials and Methods

### 2.1. Murine Strains and High-Phosphate Diet

All animal work was conducted strictly following the Guide for the Care and Use of Laboratory Animals by the National Institutes of Health. Our animal experimental protocols were approved by the Institutional Animal Care and Use Committee at the University of Texas Southwestern Medical Center.

Mice harboring floxed atg5 [43] and transgenic mice harboring GFP-LC3 reporter [44,45] were kindly provided by Dr. Noboru Mizushima (Tokyo Medical and Dental University, Tokyo, Japan). The α-MHC-Cre transgenic mouse line [46] was generously provided by Dr. Joseph Hill (UT Southwestern Medical Center, Dallas, TX, USA). The mouse strains were genotyped by polymerase chain reaction (PCR) with established protocols [43,44,45,46]. The three murine lines were intercrossed with wild-type (*WT*) 129 S1/SVlmJ (129sv) mice purchased from Jackson laboratory (Bar Harbor, ME, USA) for more than 10 generations. To generate cardiomyocyte-specific atg5 knockout (*H-atg5*^−/−^) mice, mice harboring a floxed atg5 allele were crossed with α-MHC-Cre transgenic mice. All animals were housed in a temperature-controlled room (22.0 ± 0.2 °C) with a 12:12-h light–dark cycle and were given ad libitum access to tap water and standard rodent chow containing 0.7% phosphate (*w*/*w*) (Teklad 2016, Harlan, Madison, WI, USA) unless stated otherwise. Equal male and female animals were used.

High-phosphate rodent chow (2.0% *w*/*w*) was purchased from Harlan (Teklad 08020, Harlan, Madison, WI, USA). High-phosphate chow contained 2.0% phosphorus after the addition of monobasic potassium phosphate and monobasic and monohydrate sodium phosphate. The normal-phosphate chow contained 0.7% phosphorus. Other mineral contents, including calcium (1.0%), sodium (0.2%), potassium (0.6%), chloride (0.4%), and magnesium (0.2%), were similar between high- and normal-phosphate chows. High-phosphate chow was given to *H-atg5*^−/−^ mice and *WT* mice to examine the effect of high-phosphate diet on autophagy activity and cardiac remodeling.

### 2.2. Cardiac MRI

Cardiac MRI was performed by Mouse MRI Core in Advanced Imaging Research Center and O’Brien Kidney Research Center at University of Texas Southwestern Medical Center (Dallas, TX, USA) to evaluate cardiac function and morphometry using a 7T Small Animal MR Scanner (Varian, Inc., Palo Alto, CA, USA) with a 38-mm birdcage RF coil as previously described [47,48]. Left ventricular ejection fraction and left ventricular wall thickness at systolic phase and diastolic phase were calculated using Image J software as described [48]. One investigator performed MRI acquisition and two analyzed the data independently; both were blinded to the experimental conditions (inter-operator variance < 10% was accepted). Average data from the two analysts were used.

### 2.3. Blood and Kidney Sample Collection

At predetermined times, mice were anesthetized with isoflurane and blood samples were collected in heparinized tubes and centrifuged at 3000× *g* for 5 min at 4 °C for plasma separation. At terminal studies, mice were sacrificed under anesthesia, and the kidneys were isolated, harvested, and sliced. The slices were fixed with 4% paraformaldehyde and embedded in a paraffin block or Tissue-Tek^®^ optimum cutting temperature (OCT) for histological (Hematoxylin and Eosin stain, HE, and trichrome stain, TC, USA) and immunohistologic studies, respectively.

### 2.4. Plasma Phosphate and Creatinine Determination

Plasma phosphate was measured using a Vitros Chemistry Analyzer (Ortho-Clinical Diagnosis, Rochester, NY, USA), and plasma creatinine using a P/ACE MDQ Capillary Electrophoresis System and photodiode detector (Beckman-Coulter, Fullerton, CA, USA) at the O’Brien Kidney Research Center at the University of Texas Southwestern Medical Center.

### 2.5. Heart Histopathology and Immunohistochemistry

Heart tissue was fixed in 4% paraformaldehyde (PFA) for 16 h at 4 °C, and whole hearts were cut in sagittal 4-chamber style. The 4-µm sections of paraffin-embedded heart tissue were stained with TC to evaluate cardiac fibrosis. The stained sections were examined and photographed independently by two histopathologists blinded to the experimental groups. Four-µm sections of paraffin-embedded heart or cryosections were also subjected to immunohistochemistry following the established protocols presented in our previous publications [31,49].

To quantify the cardiomyocyte cell surface as a two-dimensional surrogate parameter, paraffin-embedded sections were labeled with Alexa Fluor 555-conjugated wheat germ agglutinin (WGA) (Invitrogen, Carlsbad, CA, USA) as described previously [47,48]. Immunofluorescence images were obtained on a Zeiss 880 Confocal Microscope (Carl Zeiss Micro-Imaging Inc., Munich, Germany). Image J software was used to quantify cell surface area along the mid-chamber free wall based on WGA-positive staining [47,48,50].

To examine apoptosis in cardiomyocytes, terminal deoxynucleotidyl transferase-mediated dUTP fluorescein nick end labeling (TUNEL) assay was used to detect apoptotic cells in heart sections with an in situ cell death detection kit (Roche Diagnostics, Mannheim, Germany); nuclei were stained with DAPHOSPHATE (4,6-diamidino-2-phenylindole, DAPI) and visualized with a Zeiss LSM 880 Confocal Microscope (Carl Zeiss Micro-Imaging Inc., Munich, Germany).

All primary antibodies for immunohistochemistry were diluted in DAKO antibody diluent (S3022 Agilent, Santa Clara, CA, USA). Images were examined and acquired with the Zeiss LSM 880 Confocal Microscope system.

### 2.6. Kidney Histopathology

Kidney tissues were fixed in 4% PFA for 16 h at 4 °C, and 4 µm sections of paraffin-embedded kidney tissues were stained with Masson Trichrome (TC), respectively. Kidney histology was examined and photographed by two independent histopathologists blinded to the experimental conditions. The system to assess kidney fibrosis was generated with a previously published method. Kidney fibrosis score is expressed in arbitrary units and reflects both fibrotic area and severity of fibrosis in TC-stained sections, which were quantified with Image J software [51].

### 2.7. Immunoblot

Total heart ventricle lysate was prepared in radio-immunoprecipitation assay (RIPA) buffer containing freshly added cocktail protease inhibitors and phosphatase inhibitor cocktails (Sigma-Aldrich, St. Louis, MO, USA). Protein lysates were subjected to SDS-PAGE as described [47,48]. Filters were sequentially incubated with primary antibodies, then horseradish-peroxidase-conjugated species-specific secondary antibodies (Bio-Rad, Hercules, CA, USA), followed by enhanced chemiluminescence reaction (Bio-Rad Laboratories Inc., Hercules, CA, USA). Densitometric analyses were performed with Image J software.

### 2.8. Primary and Secondary Antibodies

The following antibodies were used for immunoblotting and/or immunohistochemistry: mouse monoclonal antibody against α-actin (MAB1501, Sigma-Aldrich, St. Louis, MO, USA); mouse monoclonal antibody against α-actinin (A7732, Sigma-Aldrich, St. Louis, MO, USA); rabbit polyclonal antibody against atg5 (NB110-53818, Novus, Centennial, CO, USA); rabbit monoclonal antibody against cleaved caspase-3 (#9661, CST, Danvers, MA, USA); rabbit polyclonal antibody against LC3 (NB100-2220, Novus, Centennial, CO, USA); mouse monoclonal antibody against p62 (H00008878-M01, Abnova, Taipei, Taiwan); mouse monoclonal antibody against α-SMA (A5528, Sigma-Aldrich, St. Louis, MO, USA).

Secondary antibodies coupled with horseradish peroxidase for immunoblotting or with fluorescein isothiocyanate, Alexa Fluor or Cy5, and Syto-61 fluorescent nuclear acid stain for immunohistochemistry were purchased from Molecular Probes/Invitrogen (Eugene, OR, USA).

### 2.9. RNA Extraction, Reverse Transcription, and Quantitative Real-Time Polymerase Chain Reaction (qPCR)

Total RNAs from left ventricles were extracted with the RNAeasy kit (Qiagen, Germantown, MD) according to the manufacturer’s protocol. Complimentary DNA (cDNA) was generated with oligo-dT primers using the SuperScript III First Strand Synthesis System (Invitrogen, Carlsbad, CA, USA) according to the manufacturer’s protocol. The qPCR primers used for mouse transcripts of α-SMA, α-actinin, β-MHC, and cyclophilin were presented in our previous publication [47]. PCR was performed in an ABI PRISM^®^ 7000 Sequence Detection System (qPCR / RT-PCR) (7000 SDS instrument) (Foster City, CA, USA) and each sample was run in triplicate. Data are expressed as amplification number of 2^−ΔΔCt^ by normalization to cyclophilin after comparison to controls using conditions from previous work [47].

### 2.10. Statistical Analysis

Quantitative data are expressed as means ± SD unless otherwise specified. Analysis was performed with SigmaPlot 13.0 software (Systat Software, Inc., San Jose, CA, USA). The normality test for all continuous variables was performed with Shapiro–Wilk, and upon failure, the variables were converted with square-root or log transformation methods when applicable, followed by regular statistical analysis. All our results were normally distributed. As appropriate, statistical analysis was performed using unpaired Student’s *t*-test or one-way or two-way analysis of variance (ANOVA), followed by Student–Newman–Keuls post-hoc test when applicable as specified. A value of *p* ≤ 0.05 was considered statistically significant.

## 3. Results

### 3.1. High Dietary Phosphate Decreases Autophagy Activity and Causes Cardiac Remodeling in WT Mice

**Figure 1:**
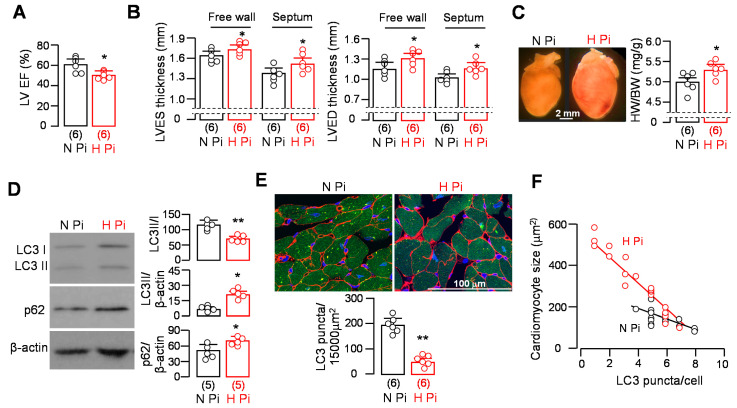
High phosphate caused cardiac remodeling and dysfunction and low autophagy activity in the hearts of *WT* mice. *WT* mice (**A**–**D**) or GFP-LC3 reporter mice (**E**,**F**) were fed normal- (N Pi) or high-phosphate (H Pi) diet for 12 weeks. After 12 weeks, mice were subjected to cardiac MRI for evaluation of cardiac function (**A**) and morphometry (**B**) followed by euthanization for heart histology study (**C**) and immunoblots (**D**). (**A**) Left ventricular ejection fraction; (**B**) left ventricular wall thickness at systole (left panel) and diastole (right panel); (**C**) heart hypertrophy in mice fed with high dietary phosphate. Left panel: representative gross appearance of heart. Right panel: heart weight/body weight. (**D**) Changes in autophagic markers in the heart. Left panel: representative immunoblots for LC3 and p62 in left ventricular lysates. Right panel: a summary of all immunoblot data. (**E**) Autophagic activity in the hearts of GFP-LC3 reporter mice. Four hours prior to sacrifice, mice were treated intraperitoneally with chloroquine (50 mg/Kg body weight). Left panel: representative micrographs of GFP-LC3 immunofluorescence puncta in free walls of left ventricles. Right panel: semi-quantitation of GFP-LC3 puncta. Sample number in each group is in brackets underneath the corresponding bars. Quantitative data are presented as mean ± SD with scatter plots of individual data points, and statistical significance was assessed by unpaired Student’s *t*-test. Significant differences are accepted when * *p* < 0.05 or ** *p* < 0.01 between two groups. (**F**) Correlation between cardiomyocyte size and the number of LC3 puncta in the hearts of LC3 reporter mice treated with normal (black symbols) or high (red symbols) dietary phosphate. The number of LC3 puncta in one heart section was counted in three locations (up, middle, and bottom) of the free wall of left ventricle; therefore, 6 mice gave 18 datasets in either the normal- or high-phosphate-fed group.

To explore whether high phosphate alters autophagy activity in the heart, we treated *WT* mice with high- (2.0%) vs. normal-phosphate chow (0.7%) for 12 weeks. We first assessed cardiac function in mice by MRI. High-phosphate-fed mice had lower left ventricular ejection fraction (Figure 1A) and thicker left ventricular wall (Figure 1B) compared with normal-phosphate-fed mice. The heart was heavier in high-phosphate-fed than normal-phosphate-fed mice (Figure 1C), which is consistent with our published data [47,52].

The effect of a high-phosphate diet on autophagic activity was reported but was inconsistent [53,54,55]. In this experiment, we first used LC3 reporter mice [31,32,56] to examine whether chronic high dietary phosphate loading alters autophagic activity in the heart. Immunoblot for LC3 and p62, molecular markers of autophagic activity, revealed that high phosphate decreased the conversion of LC3I to LC3II and increased the levels of p62 compared to normal phosphate (Figure 1D), indicating that there was low autophagy activity in mice fed with a high-phosphate diet. Immunohistochemistry confirmed low autophagy activity in heart lysates (Figure 1D) and decreased LC3 puncta in cardiomyocytes (Figure 1E). Interestingly, the lower number of LC3 puncta in larger cardiomyocytes indicated an inverse relation between the number of LC3 puncta and cardiomyocyte size. This negative correlation was more appreciable in high-phosphate-fed *WT* mice (Figure 1F), implying that lower autophagy activity may be responsible for cardiomyocyte hypertrophy.

### 3.2. H-atg5^−/−^ Mice Have Pathologic Cardiac Remodeling and Short Lifespan

**Figure 2:**
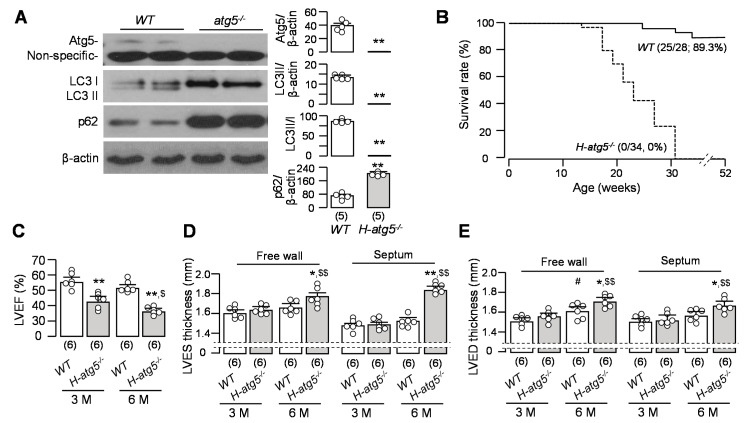
Absence of atg5 in cardiomyocytes induced short lifespan and cardiac dysfunction. (**A**) The changes in autophagic markers and atg5 in the hearts of *H-atg5*^−/−^ mice and *WT* mice at 3 months of age. Left panel: Representative immunoblots for atg5, LC3, and p62 in left ventricular lysates. Right panel: Summary of all immunoblot data. Quantitative data are presented as mean ± SD with scatter plots of individual data points, and statistical significance was assessed by unpaired Student’s *t*-test. Significant differences are accepted when ** *p* < 0.01 between two groups. (**B**) Kaplan–Meier survival curves for *H-atg5*^−/−^ and *WT* mice. (**C**) Left ventricular ejection fraction determined by cardiac MRI in *H-atg5*^−/−^ mice and *WT* mice at 3 and 6 months of age. Left ventricular wall thickness at systole (**D**) and at diastole (**E**) determined by cardiac MRI in *H-atg5*^−/−^ mice and *WT* mice at 3 and 6 months old. Sample number in each group is presented in brackets underneath corresponding bar. Quantitative data are presented as mean ± SD with scatter plots of individual data points, and statistical significance was assessed by two-way ANOVA followed by Newman–Keuls test. * *p* < 0.05, ** *p* < 0.01 vs. *WT* mice at the same age; ^#^ *p* < 0.05 vs. *WT* at 3 months old; ^$^ *p* < 0.05, ^$$^ *p* < 0.01 vs. *H-atg5*^−/−^ mice at 3 months old.

To define the consequences of atg5 deficiency in cardiomyocytes, we generated a conditional deletion of atg5 in cardiomyocytes (*H-atg5*^−/−^) and monitored cardiac function and morphology for 6 months after birth. We determined LC3 and p62 in total left ventricle lysate with immunoblot. As expected, atg5 expression was undetectable in the hearts of *H-atg5*^−/−^ mice. LC3-II levels in the hearts of *H-atg5*^−/−^ mice were extremely low, while p62 protein levels were notably higher when compared to those in *WT* mice (Figure 2A). Next, we investigated cardiac function and life span in *H-atg5*^−/−^ mice. *H-atg5*^−/−^ mice began to die at around four months of age, with no mice living longer than 9 months (Figure 2B). *H-atg5*^−/−^ mice exhibited a significant decrease in ejection fraction at ages 3 and 6 months, compared to *WT* mice (Figure 2C; Movie S1). The free wall thickness of the left ventricle and septum at left ventricular end-systole (LVES) and at left ventricular end-diastole (LVED) was significantly higher in *H-atg5*^−/−^ mice at 6 months old rather than 3 months old compared with *WT* mice (Figure 2D,E). Because of previous studies reporting that the expression of α-MHC-Cre transgene per se can induce short lifespan [57,58], we examined the heart morphology of α-MHC-Cre transgenic mice and did not see any abnormal cardiac features in α-MHC-Cre mice (data not shown) in *H-atg5*^−/−^ mice at the age of 6–9 months. Furthermore, there was a similar lifespan between α-MHC-Cre transgenic mice and *WT* mice (data not shown). Therefore, the absence of atg5 in cardiomyocytes but not the presence of α-MHC-Cre in cardiomyocytes is responsible for reduced autophagy activity, cardiac remodeling, and probably increases in cardiac mortality in *H-atg5*^−/−^ mice.

### 3.3. H-atg5^−/−^ Mice Develop Premature Cardiac Hypertrophy and Fibrosis

**Figure 3:**
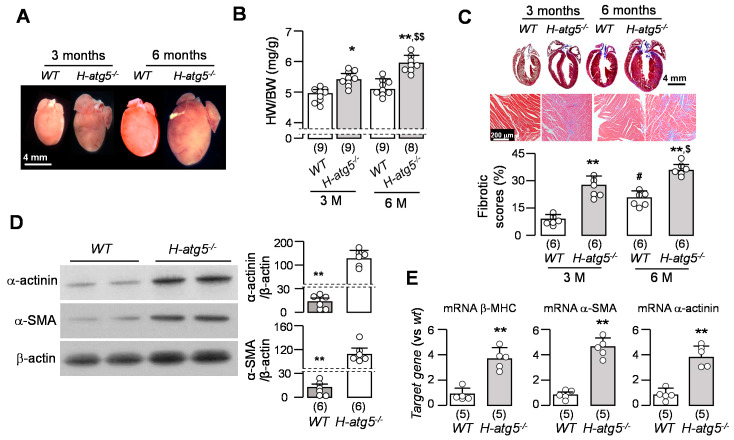
Cardiac hypertrophy and fibrosis in *H-atg5*^−/−^ mice. (**A**) Representative images of the hearts of *H-atg5*^−/−^ mice and *WT* mice at 3 and 6 months old. (**B**) Heart weight/body weight in *H-atg5*^−/−^ mice and *WT* mice at 3 and 6 months of age. (**C**) Representative macrographs (upper panel) and micrographs (middle panel) of sagittal TC-stained sections of the hearts of *H-atg5*^−/−^ and *WT* mice at 3 and 6 months of age. Semi-quantification (bottom panel) of the TC-positive area over the whole-heart section with Image J software. (**D**) Changes in hypertrophic and fibrotic markers in the hearts of *H-atg5*^−/−^ mice and *WT* mice at the age of 3 months. Left panel: representative immunoblots for α-actinin and α-SMA in left ventricular lysates. Right panel: a summary of all immunoblot data. (**E**) Quantitative analysis of transcripts of β-MHC, α-SMA, and α-actinin in left ventricular lysates in *H-atg5*^−/−^ mice and *WT* mice at the age of 3 months. Sample number in each group is presented in brackets underneath corresponding bars. Quantitative data are presented as mean ± SD with scatter plots of individual data points. * *p* < 0.05, ** *p* < 0.01 vs. *WT* mice at the same age; ^#^ *p* < 0.05 vs. *WT* at 3 months old; ^$^ *p* < 0.05, ^$$^ *p* < 0.01 vs. *H-atg5*^−/−^ mice at 3 months old by two-way ANOVA followed by Student–Newman–Keuls post-hoc test.

Hypertrophic remodeling is a common disease manifestation and a feature of age-related myocardial abnormality that can cause diastolic and/or systolic dysfunction [59]. Therefore, we tested whether defective autophagy induces hypertrophic remodeling. Consistent with previous reports [39], *H-atg5*^−/−^ mice showed increased heart volumes and weights compared to *WT* mice (Figure 3A,B). In addition, there was a marked increase in fibrosis in the hearts of *H-atg5*^−/−^ mice compared with *WT* mice (Figure 3C,D). Cardiac fibrosis was already notable in *H-atg5*^−/−^ mice at 3 months old, with no detectable cardiac fibrosis in *WT* mice at 3 and 6 months old. Furthermore, there was a significant increase in fibrotic markers (α-actinin and α-SMA) (Figure 3E), which is in agreement with the histological analysis, and the mRNA levels of α-actinin, α-SMA, and β-MHC in *H-atg5*^−/−^ mouse hearts (Figure 3F). Thus, atg5 deletion clearly led to cardiac autophagy deficiency, cardiac hypertrophy, and fibrosis. However, whether high-phosphate-induced cardiac remodeling is mediated by reduced autophagic activity is not known.

### 3.4. High Dietary Phosphate Further Enhances Mortality in H-atg5^−/−^ Mice

**Figure 4:**
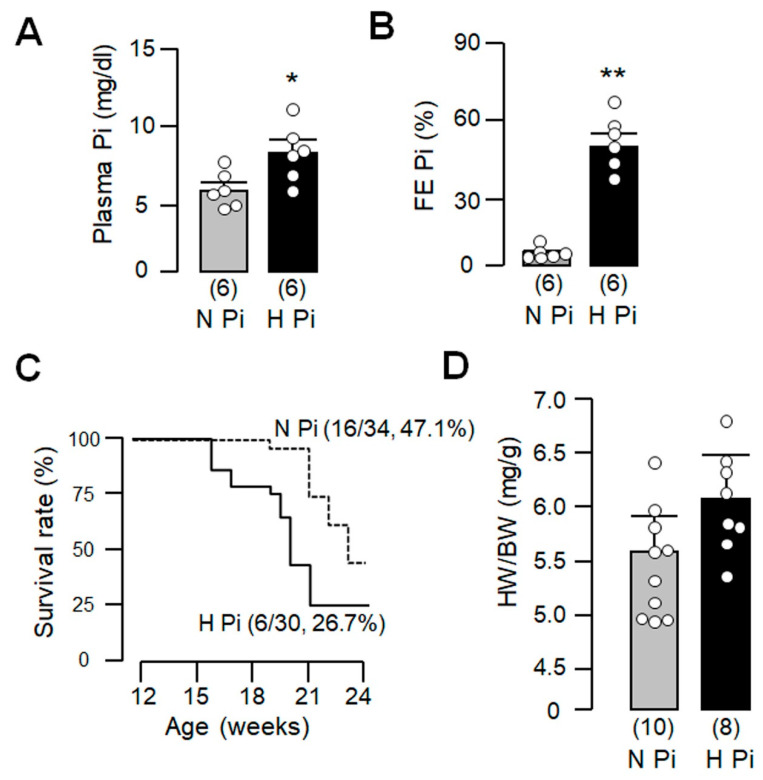
High dietary phosphate increased mortality in *H-atg5*^−/−^ mice. *H-atg5*^−/−^ mice were fed with normal- or high-phosphate diet starting at 12 weeks old for 12 weeks. (**A**) Plasma phosphate (Pi) concentration after 12-week dietary phosphate treatment in *H-atg5*^−/−^ mice; (**B**) Fractional excretion of phosphate after 12-week dietary phosphate treatment; (**C**) Kaplan–Meier survival curves of *H-atg5*^−/−^ mice fed with normal- or high-phosphate diet; (**D**) Heart weight/body weight in *H-atg5*^−/−^ mice fed with normal- or high-phosphate diet for 12 weeks. Sample number in each group is presented in brackets underneath corresponding bar. Quantitative data are presented as mean ± SD with scatter plots of individual data points, and statistical significance was assessed by unpaired Student’s *t*-test. Significant differences were accepted when * *p* < 0.05 or ** *p* < 0.01 between groups. FE_Pi_: fractional excretion of phosphate; H Pi: high-phosphate diet; N Pi: normal-phosphate diet.

Phosphotoxicity is a potential risk factor for cardiac remodeling [47,60]. To better identify whether high phosphate worsens cardiac remodeling by acting through reduced autophagy, we examined whether phosphate loading in the background of reduced autophagy still affects cardiac remodeling. We challenged *H-atg5*^−/−^ mice with a high-phosphate diet starting at 3 months of age for 12 weeks. As expected, both plasma phosphate concentration and fractional excretion of phosphate were increased after phosphate loading (Figure 4A,B). Importantly, chronic high-phosphate feeding in *H-atg5*^−/−^ mice significantly shortened the lifespan compared to *H-atg5*^−/−^ mice fed a normal diet. The 12-week survival of *H-atg5*^−/−^ mice was 47.1% (16/34) given normal dietary phosphate and was reduced to 26.7% (6/30) with high dietary phosphate feeding (Figure 4C). The ratio of heart weight over body weight was significantly increased in mice fed high dietary phosphate (Figure 4D). The fact that high dietary phosphate shortened the lifespan even in the presence of already reduced autophagy in the heart in *H-atg5*^−/−^ mice suggests that phosphate toxicity is mediated independently of atg5 and autophagy.

### 3.5. The Additive Effect of High Phosphate and Atg5 Deficiency in Pathological Cardiac Remodeling

**Figure 5:**
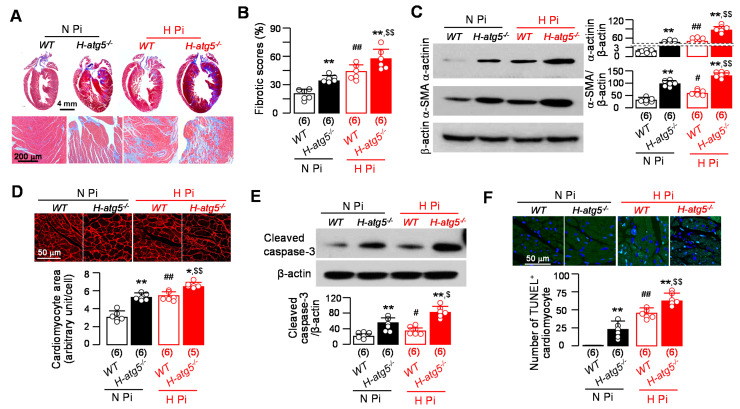
High phosphate exacerbated pathological cardiac remodeling in *H-atg5*^−/−^ mice. *WT* mice or *H-atg5*^−/−^ mice were fed with normal- or high-phosphate diet starting at 3 months old for 12 weeks. (**A**) Representative macrographs (upper panel) and micrographs (bottom panel) of sagittal TC-stained sections of the hearts. (**B**) Semi-quantification of the TC-positive area over the whole-heart section with Image J. (**C**) Changes in hypertrophic and fibrotic markers in the heart. Left panel: representative immunoblots of left ventricular lysates for α-actinin and α-SMA protein. Right panel: a summary of all immunoblot data. (**D**) Hypertrophic cardiomyocytes in free walls of left ventricles of mice. Upper panel: representative immunohistochemistry for WGA. Bottom panel: semi-quantification of myocyte size with Image J. (**E**) The changes in apoptotic markers in the heart. Upper panel: representative immunoblots for cleaved caspase-3 in left ventricular lysates. Bottom panel: a summary of immunoblot data. (**F**) Changes in apoptotic markers in the heart. Upper panel: representative immunofluorescent images for TUNEL in free wall of left ventricles. Bottom panel: semi-quantification of TUNEL-positive cells/DAPI-positive cardiomyocytes. Sample number in each group is presented in brackets underneath corresponding bars. Quantitative data are presented as mean ± SD with scatter plots of individual data points. * *p* < 0.05, ** *p* < 0.01 vs. *WT* mice at the same dietary phosphate treatment; ^#^ *p* < 0.05, ^##^ *p* < 0.01 vs. *WT* treated with normal-phosphate diet; ^$^ *p* < 0.05, ^$$^ *p* < 0.01 vs. *H-atg5*^−/−^ mice treated with normal-phosphate diet. Statistical significance was assessed by two-way ANOVA followed by Newman–Keuls test. H Pi: high-phosphate diet; N Pi: normal-phosphate diet.

High phosphate suppresses autophagy and low autophagy leads to pathologic cardiac remodeling. The critical question is whether phosphate-induced cardiomyopathy [47,52] is mediated through impaired autophagy. To explore whether high phosphate can worsen cardiac remodeling in the background of already impaired autophagy in *H-atg5*^−/−^ mice, we fed *WT* and *H-atg5*^−/−^ mice a high-phosphate diet and compared them to those fed a normal-phosphate diet. A four-way comparison was made. In Trichrome-stained heart sections (Figure 5A), there was more fibrosis in the hearts of *H-atg5*^−/−^ mice compared to *WT* mice both on a normal-phosphate diet. High dietary phosphate-fed *WT* mice had more fibrosis in the heart compared to *WT* mice fed with normal dietary phosphate. The most important finding is that high dietary phosphate led to a significant increase in cardiac fibrosis in *H-atg5*^−/−^ mice compared to normal phosphate (Figure 5A,B). The levels of two cardiac fibrosis markers, α-SMA and α-actinin, were significantly increased in high dietary phosphate-fed *H-atg5*^−/−^ mice (Figure 5C), accompanied by cardiomyocyte hypertrophy (Figure 5D).

High phosphate is known to induce cell apoptosis [61,62]. The current study confirmed this model as there was increased active caspase-3 protein expression, an apoptotic marker in heart lysates (Figure 5E), and more TUNEL-positive cardiomyocytes and apoptotic cardiomyocytes (Figure 5F) in *WT* mice fed with high dietary phosphate compared to those fed normal dietary chow. Moreover, active caspase-3 protein expression was increased in the heart lysates of in *H-atg5*^−/−^ mice compared to *WT* mice under normal dietary chow, suggesting that low autophagy increases apoptosis in the heart (Figure 5E). The high-phosphate-induced elevated apoptosis in the heart was exaggerated in *H-atg5*^−/−^ mice (Figure 5E). We found that high dietary phosphate increased TUNEL staining in the cardiomyocytes of *WT* mice (Figure 5F). At baseline, under normal dietary phosphate, *H-atg5*^−/−^ mice had higher numbers of TUNEL-positive cardiomyocytes than *WT* mice. High dietary phosphate increased apoptotic cardiomyocytes in both *WT* mice and *H-atg5*^−/−^ mice. The number of apoptotic cells was significantly increased in *H-atg5*^−/−^ mice compared to *WT* mice on high-phosphate chow (Figure 5F). Most importantly, high phosphate significantly increased apoptotic cells in the background of low autophagy in the *H-atg5*^−/−^ mice. Therefore, high dietary phosphate exacerbates cardiac remodeling and cardiac dysfunction beyond and above atg5.

### 3.6. High Dietary Phosphate Induces Similar Kidney Damage between WT Mice and H-atg5^−/−^ Mice

**Figure 6:**
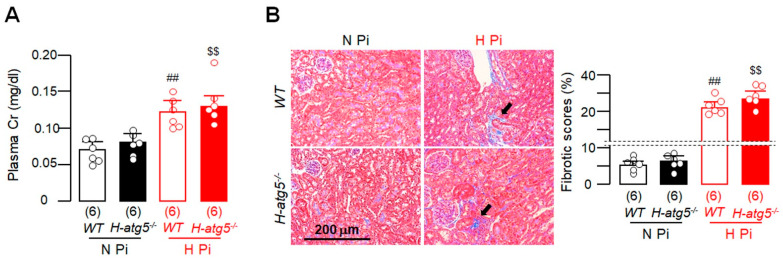
High phosphate induced similar severity of kidney fibrosis between *WT* and *H-atg5*^−/−^ mice. *WT* and *H-atg5*^−/−^ mice were fed with normal- or high-phosphate diet starting at 3 months old for 12 weeks. (**A**) Plasma creatinine (Cr). (**B**) Kidney fibrosis was determined in TC-stained sections. Left panel: Representative micrographs of TC-stained kidney sections. Black arrows depict tubulointerstitial fibrosis. Right panel: Semi-quantification of the TC-positive area over the kidney section with Image J. Sample number in each group is presented in brackets underneath corresponding bar. Quantitative data are presented as mean ± SD with scatter plots of individual data points. ^##^ *p* < 0.01 vs. *WT* treated with normal-phosphate diet; ^$$^ *p* < 0.01 vs. *H-atg5*^−/−^ mice treated with normal-phosphate diet. Statistical significance was assessed by two-way ANOVA followed by Newman–Keuls test. H Pi: high-phosphate diet; N Pi: normal-phosphate diet.

The heart and the kidneys are functionally interdependent and the reciprocal detrimental interaction between kidney and heart disease is collectively called cardiorenal syndrome [63,64]. We examined the possibility that high-phosphate-induced kidney damage in *H-atg5*^−/−^ mice may be more severe than in *WT* mice and thus provide a possible explanation for the results described above. Interestingly, kidney function based on plasma Cr (Figure 6A) and kidney fibrosis assessed by Trichrome stain (Figure 6B,C) were similar in *H-atg5*^−/−^ mice and *WT* mice after long-term high dietary phosphate loading. This indicates that the more severe pathologic cardiac remodeling in *H-atg5*^−/−^ mice fed with high dietary phosphate did not result from worse kidney damage in *H-atg5*^−/−^ mice.

## 4. Discussion

High phosphate is a contributor to aging and age-associated degeneration in multiple organs [8,31,51,65]. However, the cellular and molecular mechanisms of phosphotoxicity are complicated and not completely defined. Our previous studies showed that high phosphate downregulates autophagy activity in the kidney [31,32], and low autophagy activity in the kidney exacerbated ischemia kidney injury [49,66] and accelerated acute kidney injury transition to chronic kidney disease [44]. The current study provides in vivo evidence to support the notion that high phosphate impairs autophagy activity in the heart and promotes cardiac remodeling. In addition, in the presence of impaired cardiac autophagy, high phosphate still worsens cardiomyopathy, indicating that there are autophagy-independent pathways by which phosphotoxicity is mediated in the heart.

*Atg5 deletion in cardiomyocytes induces pathologic cardiac remodeling:* Overwhelming experimental evidence supports the notion that dysregulated autophagy is a crucial driving force in cardiac aging and pathology [67]. Global atg5 knockout mice have embryonic or neonatal death [68] and cardiac-specific atg5 deficiency led to cardiac hypertrophy, left ventricular dilatation, and contractile dysfunction [39]. Consistent with previous reports, our results showed that mice with cardiomyocyte-specific deletion of atg5 developed cardiac dysfunction, hypertrophy, and fibrosis at 6 months of age and no mice lived longer than 9 months. It has been shown that some α-MHC-Cre mice had shorter lifespans than *WT* littermates, suggesting a negative effect of α-MHC-driven Cre recombinase expression in the heart [57,58]. The α-MHC-Cre mice used in this study had normal cardiac histology and morphology and normal lifespan, so it is justified to conclude that the abnormal cardiac phenotype in *H-atg5*^−/−^ mice results from the absence of atg5 and not from the presence of the α-MHC-Cre transgene.

*Long-term dietary phosphate loading induces autophagy deficiency in the heart:* Whether high phosphate modulates autophagy activity is controversial. High phosphate is associated with upregulated autophagy-related protein expression and autophagy activity in cultured cells and in rodents [42,53,54,69]. However, suppression of autophagy by high phosphate has also been noted [31,41,55]. In cultured vascular smooth muscle cells, calcium deposition was increased by autophagy inhibition and decreased by autophagy induction [40]. Increased levels of extracellular phosphate activated the mTORC1 pathway, a well-known autophagy inhibitory signaling cascade [70], and consequently reduced autophagy [69]. We found that mice fed a high-phosphate diet had reduced autophagy activity in cardiomyocytes. The lower number of LC3 puncta in cardiomyocytes in the larger cardiomyocytes further supports the causal relationship between autophagy and cardiomyocyte hypertrophy.

*Potential mechanisms of downregulation of autophagy activity by phosphate:* The current study did not seek to define the cellular and molecular mechanisms by which high phosphate modulates autophagy activity in the heart. Our previous work in the kidney suggests that high phosphate downregulates autophagy activity in kidney tubules through direct and indirect mechanisms: (1) promotion of beclin 1 binding to its negative regulator bcl2; (2) impairment of lysosomal–autolysosomal degradation; (3) downregulation of Klotho, which further decreases autophagy activity [31]. The current proof-of-concept study showed that 12-week high dietary phosphate feeding increased plasma phosphate and decreased kidney and circulating Klotho in mice, which is compatible with our previous hypothesis [31].

**Figure 7:**
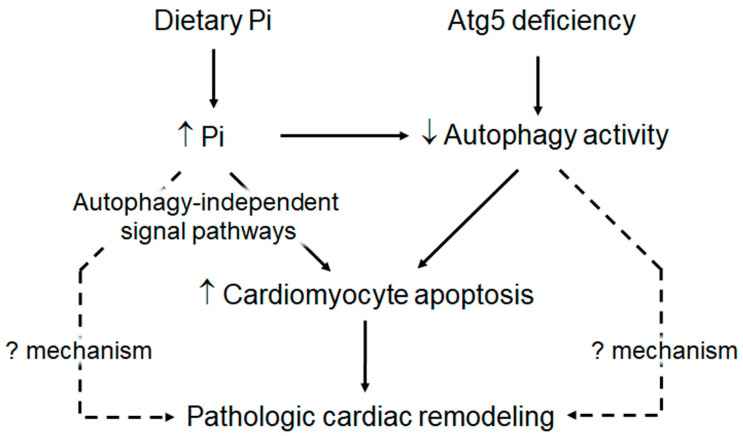
High phosphate exacerbates atg5 deficiency-induced cardiac remodeling. High phosphate decreases autophagy activity in cardiomyocytes. Atg5 deletion in cardiomyocytes downregulates autophagy activity in cardiomyocytes. Low autophagy activity results in cardiomyocyte apoptosis. High phosphate also induces cardiomyocyte apoptosis, which is autophagy-independent. Increase in apoptosis in cardiomyocytes leads to cardiomyocyte damage and cardiac hypertrophy and fibrosis. Therefore, high phosphate and low autophagy individually trigger and additionally promote heart deterioration. However, high-phosphate-induced pathologic cardiac remodeling might be autophagy-independent and apoptosis-independent (dash line), whose mechanisms are not explored in the current study. On the other hand, low autophagy can induce cardiac remodeling independently of apoptosis (dash line), which is worth illustrating.

*Long-term dietary phosphate loading accelerates pathologic cardiac remodeling and enhances premature death in H-atg5^−/−^ mice:* The current results are consistent with the phosphotoxicity in the heart in rodents with normal or abnormal kidney function [47,48,52,71]. There are two important novel findings in this study. First, *H-atg5*^−/−^ mice fed a high-phosphate diet had higher mortality and cardiac fibrosis progression compared to *WT* mice fed high-phosphate chow at the same age. Second, high dietary phosphate-fed *H-atg5*^−/−^ mice still had higher mortality and worse cardiac remodeling compared to *H-atg5*^−/−^ mice fed with normal dietary phosphate, indicating that high phosphate induces cardiac toxicity independently of autophagy (Figure 7). Therefore, the additive effect of high phosphate and Atg5 deficiency indicates that reduced autophagy is not the sole mechanism by which phosphate induces pathologic cardiac remodeling.

*Cardiac phosphotoxicity is a result of abnormal cell fate:* Both autophagy and apoptosis are cellular processes that regulate cell survival and death. Autophagy provides cellular quality control by eliminating dysfunctional components such as damaged organelles [72,73]. Apoptosis is programmed cell death aimed at controlling cell fate by constraining injured cells to minimize damage to surrounding tissues and prevent necrosis, a type of cell death following a severe insult, resulting in spillage of the contents of the cell into surrounding tissues, amplifying damage [72,73]. However, over-active apoptosis also leads to function loss and is considered a cause of cardiac remodeling in the chronically overloaded [74], ischemic [75,76], and aging heart [77].

Our study showed a greater degree of apoptosis in the hearts of *H-atg5*^−/−^ mice than *WT* mice without phosphate challenge, implying that atg5 absence in cardiomyocytes activates apoptotic machinery through a yet-to-be determined mechanism. Apoptosis does not function alone in determining cell fate. Autophagy engages in a complex interplay with apoptosis [78,79]. Generally, autophagy can prevent the activation of apoptotic pathways through the removal of, for example, damaged mitochondria [80]. On the other hand, autophagy deficiency increases apoptosis and cell death [39,81] because low autophagy fails to re-establish cellular homeostasis for cell survival [78,79,82,83].

High phosphate induces apoptosis in cultured odontoblasts [84], endothelial cells [85], and vascular smooth muscle cells [86]. We also found that high phosphate induces apoptosis in the heart, suggesting that high-phosphate-induced apoptosis might be one of the general cellular mechanisms of phosphotoxicity. Normalization of serum phosphate increases autophagy activity and reduces apoptosis [62,86], indicating that downregulation of autophagy activity may be a mediator of high-phosphate-induced apoptosis. The finding of more apoptosis in cardiomyocytes in high-phosphate-fed *H-atg5*^−/−^ mice compared to normal-phosphate-fed *H-atg5*^−/−^ mice or high-phosphate-fed *WT* mice suggests that apoptosis in cardiomyocytes may be the primary target of high-phosphate damage. Further studies are required to determine the underlying molecular mechanism for high-phosphate-induced apoptosis.

There are some limitations in the current study. First, we showed that high phosphate exacerbates cardiac phenotypes in atg5-deficient mice, suggesting that high phosphate induces cardiomyopathy through an atg5-independent pathway. We need to explore whether the upregulation of atg5 activity in cardiomyocytes could attenuate cardiac phosphotoxicity. Second, our previously published data showed that Klotho [87] attenuated cardiomyopathy induced by high phosphate and global autophagy deficiency [31,47]. We also found that Klotho upregulated autophagy activity and rescued phenotypes, including the reduction of plasma phosphate and attenuation of ischemia-induced kidney damage in autophagy-deficient mice [31,44,49]. The current study did not examine Klotho’s effect on cardiac phenotypes in mice harboring an atg5 deletion, although we assumed that Klotho deficiency might contribute to cardiac remodeling in high-phosphate diet and atg5 deletion based on our previous publication [31,32]. The cardiac impact of Klotho merits further investigations in atg5-deficient mice.

## 5. Conclusions

We demonstrated that high dietary phosphate loading caused cardiac hypertrophy and fibrosis in *WT* mice accompanied by a decrease in autophagic activity and increased apoptosis in the heart. Mice with a conditional deletion of atg5 in cardiomyocytes have a shorter lifespan, higher level of apoptosis in cardiomyocytes, severe cardiomyopathy characterized by cardiac dysfunction, hypertrophy, and fibrosis. A key finding is that a high-phosphate diet further exacerbated cardiac remodeling and shortened the lifespan in *H-atg5*^−/−^ mice, independent of kidney damage. Therefore, high phosphate and low autophagy individually trigger and amplify a vicious vortex to induce apoptosis in heart tissue and promote heart pathology (Figure 7). In summary, high phosphate interplays with autophagy deficiency in the induction of apoptosis in cardiomyocytes, and the initiation and acceleration of pathologic cardiac remodeling, which is relevant for the high morbidity and mortality of cardiovascular diseases in the aging population. Upregulation of autophagy or/and control of plasma phosphate are potential strategies for the treatment of metabolic cardiomyopathy.

## Data Availability

Not applicable.

## Supplementary Materials

The following are available online at https://www.mdpi.com/article/10.3390/cells10040777/s1. Movie S1. Cardiac magnetic resonance imaging (MRI) in *WT* mice and *H-atg5*^−/−^ mice at 3 months old. Cardiac MRI movies were made with Image J software.
