# Supplementary material for: High Dietary Phosphate Exacerbates and Acts Independently of Low Autophagy Activity in Pathological Cardiac Remodeling and Dysfunction
Source: Cells. 2021 Apr 1;10(4):777. doi: 10.3390/cells10040777 (PMC8065663; doi:10.3390/cells10040777)
Supplement: Supplementary file 1 [file cells-10-00777-s001.zip › cells-1158215-supplementary.pptx]

## Slide 1
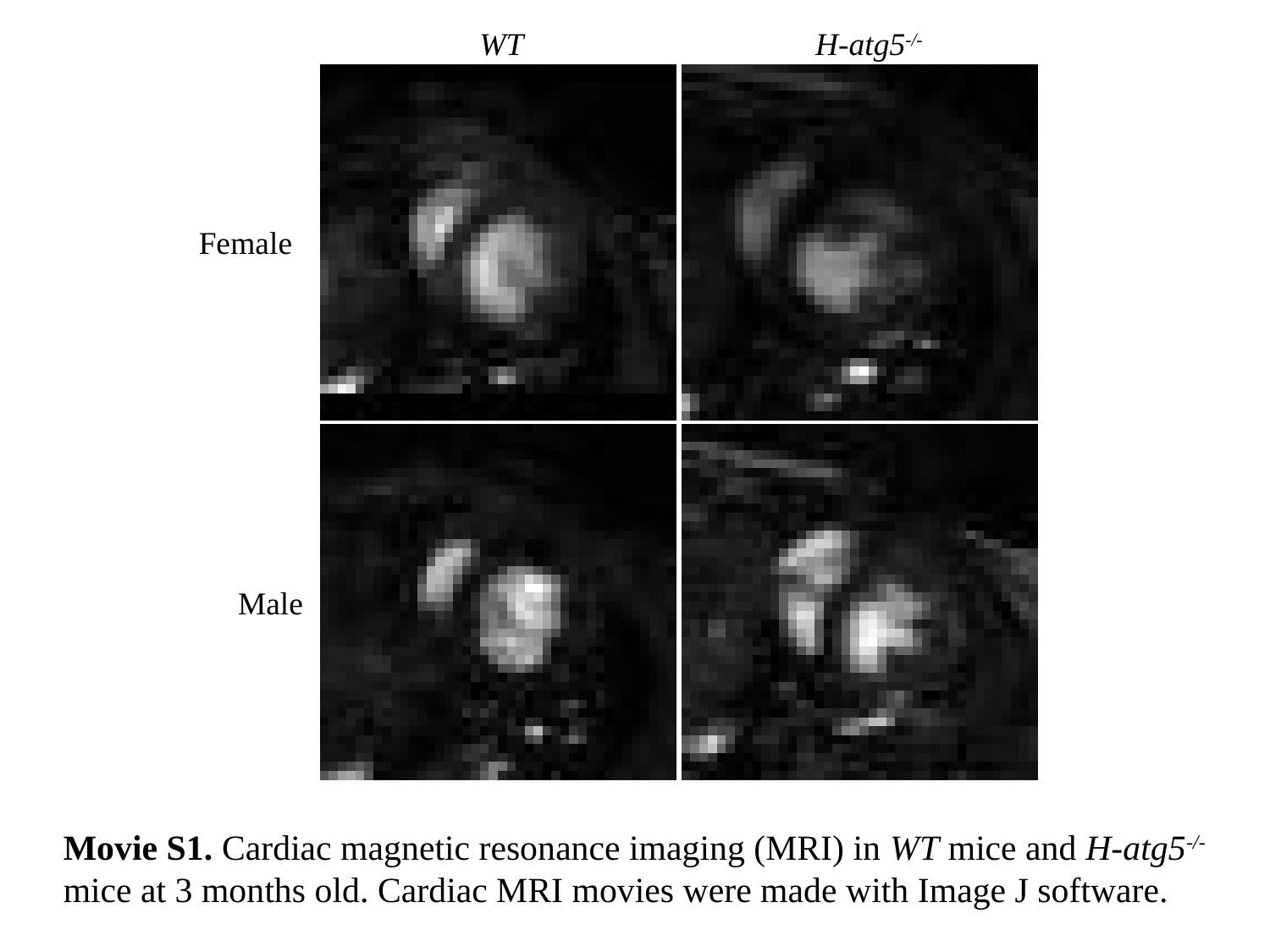

WT
H-atg5-/-
Female
Male
Movie S1. Cardiac magnetic resonance imaging (MRI) in WT mice and H-atg5-/- mice at 3 months old. Cardiac MRI movies were made with Image J software.
